# Supplementary material for: Expression of a Large Single-Chain 13F6 Antibody with Binding Activity against Ebola Virus-Like Particles in a Plant System
Source: Int J Mol Sci. 2020 Sep 23;21(19):7007. doi: 10.3390/ijms21197007 (PMC7582593; doi:10.3390/ijms21197007)
Supplement: Supplementary file 1 [file ijms-21-07007-s001.pdf]

International Journal of
Molecular Sciences

Expression of a Large Single-Chain 13F6 Antibody with Binding Activity Against Ebola Virus-Like Particles in a Plant System

Sohee Lim and Do-Sun Kim, Kisung Ko

1. Supplementary Figures

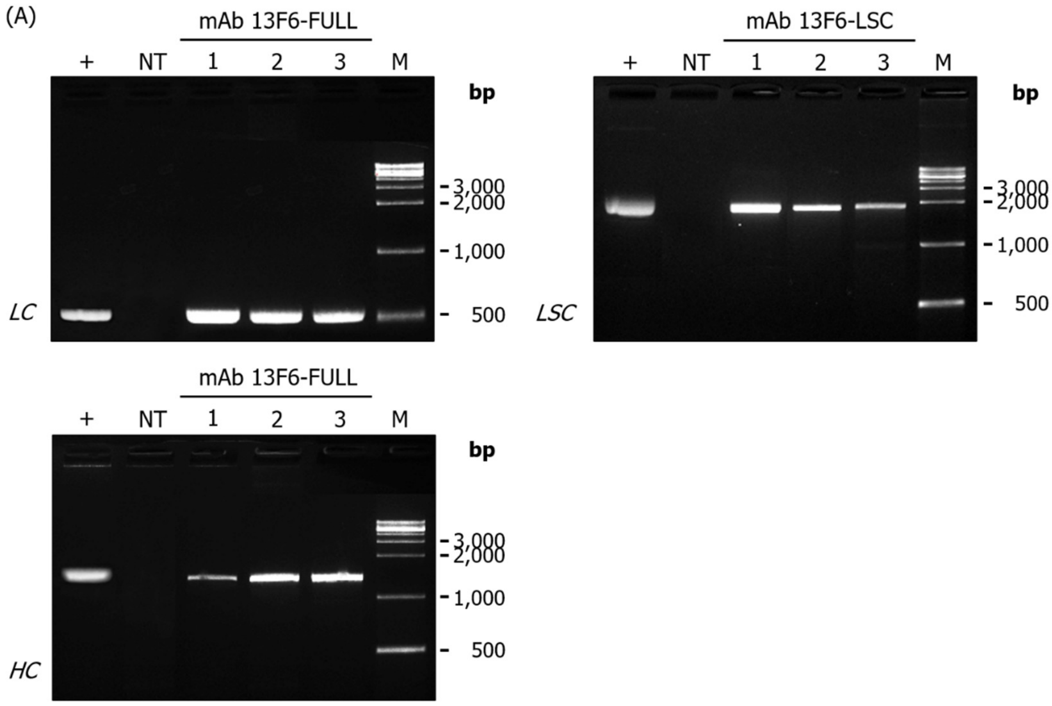

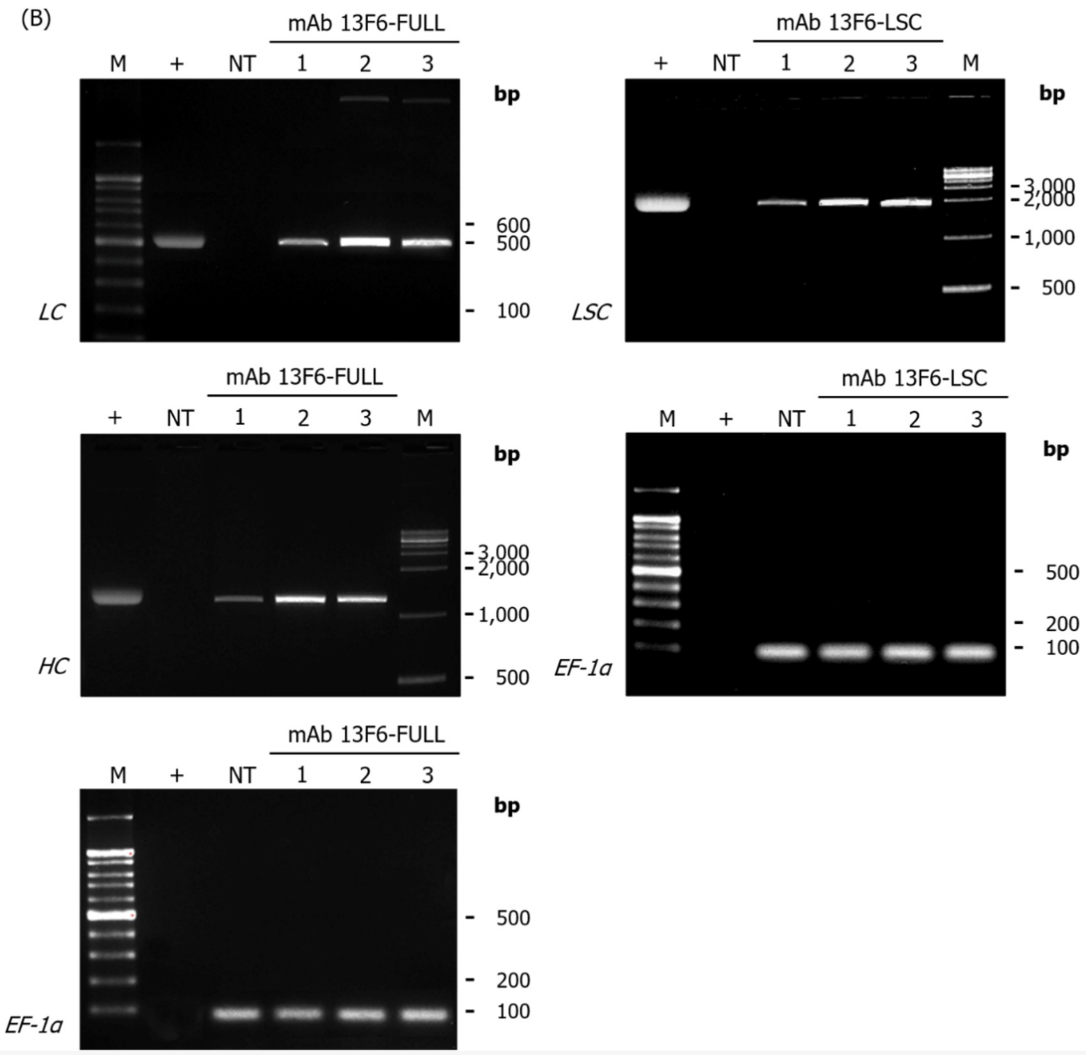

Figure S1. Entire gels of electrophoresis for Figure 2. PCR and RT-PCR analyses of transgenic LC, HC, and LSC in gDNA and mRNA of the transgenic plant expressing mAbs 13F6-FULL and 13F6-LSC. (A) PCR analysis was performed to confirm the existence of LC and HC of mAb 13F6-FULL and LSC of mAb 13F6-LSC gene construct in the plant transformants. (B) RT-PCR analysis was conducted to confirm the mRNA transcription of LC and HC of mAb 13F6-FULL and LSC of mAb 13F6-LSC gene construct in the plant transformants. The relative transcription level of each gene was normalized by using the *EF-1α* gene as the internal control. Numbers on the right indicate size marker (bp). M: DNA size marker; +: positive control, pBIN 13F6-FULL and pBI 13F6-LSC in DH5α; NT: negative control, non-transgenic *N. tabacum* plant; 1-3: Transgenic line #. The volume of sample loaded was 10 μL for each sample.

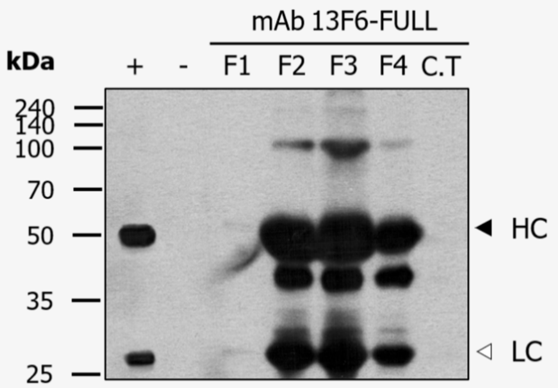

Figure S2. Immunoblot analysis to confirm the purified fraction of mAb 13F6-FULL from transgenic *N. tabacum* plants. An immunoblot analysis was performed to identify the HC and LC of purified mAb 13F6-FULL proteins from transgenic plants. The purified mAb 13F6-FULL was detected by the goat anti-human Fc fragment specific antibody and the goat anti-human IgG Fab fragment specific antibody, respectively. Numbers on the left indicate molecular weight (kDa). +: commercial 13F6 antibody (mAb 13F6-C) as the positive control; NT: non-transgenic *N. tabacum* plant as the negative control; F1-F4: purified samples from transgenic plant expressing mAb 13F6-FULL; C.T, column through. Each well had 20 μL of the sample loaded in it. Black and white arrow heads indicate HC, and LC protein bands, respectively.
